# Antitubercular Activity and Isolation of Chemical Constituents from plant *Vitex negundo* Linn

**Published:** 2018

**Authors:** Padma Laxmikant Ladda, Chandrakant Shripal Magdum

**Affiliations:** a *Department of Pharmacology, Appasaheb Birnale College of Pharmacy, Sangli.Maharastra, India. *; b *Department of Chemistry, Rajarambapu College of Pharmacy, Kasegaon Maharastra, India.*

**Keywords:** Mycobacterium tuberculosis, Vitex negundo, NRA, HPTLC, Ursolic acid

## Abstract

Tuberculosis still remains a leading cause of death in the world. Currently considerable interest in natural products and their derivatives in the area of drug research for multidrug resistant tuberculosis (MDR-TB). The present investigation focused on identification, isolation, characterization of lead constituents and to determine the antitubercular activity of their enriched fractions and isolated compounds by Nitrate reductase assay (NRA) method. Leaves extracted with ethanol by soxhlet extraction and ethanol extract separated in petroleum ether, chloroform and methanol by separating funnel and fractionated by column chromatography. Ethanol extract, petroleum ether and chloroform fraction showed antitubercular activity at 150 µg/mL. Isolated HEA-2, CM-20 and CM-24 showed MIC at 100 while PE-34 at 50 and 100 µg/mL. β-sitosterol content in chloroform and petroleum ether fractions was calculated by using HPTLC. Pet. ether and chloroform fractions of ethanol extract which contains betulinic acid, ursolic acid and β -sitosterol shows anti- TB activity. HPTLC, IR, 1 H NMR and GC-MS study of isolated PE-34 gave satisfactory results for confirmation of the structure as ursolic acid with significant antitubercular potential.

## Introduction

Tuberculosis is a major threat killing about 2 million people each year. WHO estimates that 1 billion people will be newly infected in the period 2000-2020, resulting in 35 more million deaths, nearly one billion more people will be newly infected, 200 million will get sick and 70 million will die from TB if control not strengthened and active TB left if untreated (1, 2). HIV out-breaks; India can have an additional impact on the increase of TB in India. The human immune deficiency virus (HIV) infection, MDR-TB, poverty, migration, ethnic conflicts, and substance abuse is an increasing problem and has become an additional challenge to TB control efforts (3, 4). A combination of factors has contributed to the observed increase in tuberculosis cases, including the worldwide*. *The situation has recently been complicated by the human immunodeficiency virus (HIV) pandemic and the increased prevalence of multi-drug resistant strains of *Mycobacterium tuberculosis* (5). In spite of the availability of effective antitubercular drugs, such as isoniazid and rifampin, the emergence of resistant strains of *M. tuberculosis*, the pathogenic synergy of the tubercular and nontubercular mycobacterial infections with HIV infections the scarce compliance with the complex therapeutic regimens, justify the effort directed to the investigation of new drugs for the treatment of tuberculosis and other atypical mycobacterioses (6).

Natural products, or their semi-synthetic derivatives, well defined as providing novel examples of anti-infective drug leads, currently play important roles in the chemotherapy of tuberculosis (7). The urgent need to create or find new drugs to reduce the global burden of tuberculosis is much discussed in the current biomedical literature (8). Plant *Vitex negundo* Linn. (Family: Verbenaceae) commonly known as Nirgundi. It grows gregariously in wastelands and is also widely used as a hedge-plant (9). The decoction of leaves is used for treatment of inflammation, eye-disease, toothache, leucoderma, enlargement of the spleen, ulcers, cancers, catarrhal fever, rheumatoid arthritis, gonorrhea, sinuses, scrofulous sores, bronchitis and as tonics (10). The leaves of *Vitex negundo *Linn are antibacterial, laxative, antioxidant, anticonvulsant, hypoglycemic, and anti-inflammatory properties (11-17). The juice of the leaves is used for removing foetid discharges and worms from ulcers, whilst oil prepared with the leaf juice is applied to sinuses and scrofulous, vermifuge, pain reliever, and insect repellents sores. Extracts of the leaves have shown bactericidal and antitumor activity and leaves are anti-parasitical (18, 19).

The challenge of discovering new, urgently needed antitubercular drugs from natural sources requires a truly interdisciplinary research. Innovative natural products chemistry tools have to be developed and employed in order to meet these demands (20). In earlier papers we have reported the antitubercular activity and the phytochemicals of the ethanol and other different extracts of *Vitex negundo *Linn (21). Despite its popular use as a medicinal plant, no data have been published on the antitubercular activity of chemical constituents of leaves extracts of this species. The present study reports the antitubercular activity of various column fractions obtained from leaf extracts of this plant and isolation and identification of some active natural leads structure for future 

studies.

## Experimental

*General*

The purified compounds were subjected to NMR (Bruker model AV 300MHZ^1^H- NMR spectra in CDCl_3 _were acquired with a Varian INOVA 500 spectrometer, operating at 300 MHz for  Chemical shifts were given in δ(ppm), using tetramethylsilane (TMS) as internal standard. GC-MS was recorded on (Hewlett Packard, GCD: 1800 A.) For identification FT-IR (Jasco FT-IR-410), UV-spectrophotometer (Jasco V550), HPTLC system (Camag with Wincat, Anchrome lab, Mumbai) used in study. β-sitosterol purchased from Sigma-aldrich, Mumbai, India.

The chromatography separations were performed using columns chromatography with silica gel 60 (60-120 mesh, Merck) and thin layer chromatography (TLC) using merck silica gel 60 F254 pre-coated plates (0.25 mm). All other chemicals and regents used in the work were of analytical grade and purchased from (E Merck, Mumbai) India. Loweinstein-Jensen media and McCartney bottles purchased from Himedia, Mumbai. BacT/Alert 3D system (Biomerieux, France) used for evaluation of antitubercular activity.* Mycobacterium tuberculosis H37RV *procured from Jalma institute of Leprosy and other mycobacterial diseases, Agra (India) 

*Plant material*

Fresh leaves of the plant *Vitex negundo *Linn were collected from Sangli, and Miraj areas (MS,India) in Nov to Dec. 2009. Plant specimen was botanically identified by the botanist, Smt. U. S. Shinde, Department of botany, Willingdon College, Sangli India. A voucher specimen has been deposited at the herbarium in the department of Botany, Shivaji university Kolhapur. 

*Extraction and phytochemical analysis*

Leaves were cleaned with deionized water, air dried, and powdered in a blender. Powder material was extracted by soxhlet extractor with ethanol separately and sequentially extracted with different solvents according to their increasing polarity (petroleum ether, benzene, chloroform, methanol and water). The extracts were subjected to preliminary phytochemical tests and reported its results earlier in research paper (21). 

*Evaluation of antitubercular activity*

Antitubercular screening was carried out against *Mycobacterium tuberculosis H37RV *by NRA and other different methods. 150 µg/mL of ethanol extract, 200 µg/mL of petroleum ether extract and chloroform extract of *Vitex negundo* Linn. was reported antitubercular activity earlier in research paper (21). Screening of antitubercular activity of petroleum ether, chloroform and methanol fractions of ethanolic extract were performed by NRA (21-23) and BacT/Alert method (21, 24, 25). Antitubercular activity was performed for all isolated compounds by NRA method.

In the present study, NRA which is based on the ability of *M. tuberculosis *to reduce nitrate to nitrite, the reduction detected by using specific reagents which produces a colour change. The critical concentrations used were 0.2µg/mL for Isoniazid, 40µg/mL for rifampicin standard and different concentrations of extract samples of the plant. 

The LJ media prepared according to procedure was described in proportion method (26). The Golyshevskaia *et al. *and Angeby *et al.* reported the concentration (KNO_3_- 1 mg/mL) used in the media. However growth was not observed of *M. tuberculosis *H37RV (in the form of pink colour) in control bottles. Hence, the method was modified as potassium nitrate (KNO_3_- 30 mg/mL) added to the media and the growth was observed of *M. tuberculosis *H37RV (in the form of pink colour) in control bottles.

In all control bottles and methanol fraction reddish/violet coloration was observed on the surface of the slants indicative of a positive NRA. No reddish/violet coloration was observed on the surface of the slants for rifampicin, INH, petroleum ether fraction and chloroform fraction of ethanol extract, absence of colouration interpreted as negative NRA. The results were obtained after seven days of inoculation in all the control and test sample containing bottles.

BacT/Alert method was used according to procedure described by Ladda *et al.* (21). In petroleum ether fraction and chloroform fraction of ethanol extract of *Vitex negundo* Linn. at 150µg/mL observed the growth of *M. tuberculosis *later than proportional control bottle (after 10.38 days) after inoculation. Growth of *M. tuberculosis *identified by yellow colour at the bottom of bottle and indicated +ve signal which was also confirmed by acid fast staining. Therefore, petroleum ether and chloroform fraction of ethanol extract showed antitubercular activity since the growth of *M. tuberculosis *in each test bottle was later than the proportional control bottle by BacT/Alert method.

Petroleum ether extract and Chloroform extract gave positive identification tests for the Libermann-Burchard reaction for sterols. Literature reported that triterpenes were mostly soluble in petroleum ether and chloroform and triterpenes have antitubercular activity (8). As ethanol extract shows potential antitubercular activity and on the basis of phytochemical analysis and TLC examination, we chose the ethanol extract for column chromatography. Hence, separation of phytoconstituents from ethanol extract in petroleum ether, chloroform and methanol was carried out by separating funnel and collected each solvent fraction further subjected for separation and isolation of phytoconstituents by column chromatography method. 

*Column chromatographic separation*

The ethanol extract was subjected to column chromatography over silica gel (130g, 60-120 mesh). Elution of the column was performed using graded mixture of n-hexane and ethyl acetate (4:1 v/v). Different 15 fractions of elution based on time were collected and tested by TLC and spots were detected by Anisaldehyde-sulphuric acid and calculated Rf value. Similar fractions were based on their similarity in composition, deduced from TLC analyses combined and labelled as HEA-1 and 2. 

*Column chromatography of chloroform fraction of ethanol extract*

The column was eluted with graded mixture of chloroform and methanol. A total of 40 fractions were collected and TLC of each fraction was carried out to find Rf and detected with anisaldehyde-sulphuric acid for the separations of triterpenes. Fractions showed same identical λmax and Rf value in mobile phase chloroform: methanol (15:1), therefore it was combined and labelled as CM-12, CM-13 CM-20 and CM-24. 

Column chromatography of petroleum ether fraction of ethanol extract was performed by two methods as follows: 

A] The column was eluted with graded mixture of chloroform and methanol (50, 49.5:0.5, 49.4:0.6, 49.3:0.7, 49.2:0.8, 49.1:0.9, and 50 mL). Similar fractions were combined together and labelled as PE-9, PE-10, PE-19, and PE-34. 

B] another column was eluted with graded mixture of petroleum ether: ethyl acetate (4: 1), fractions which showed similar Rf value and λ max in mobile phase petroleum ether: ethyl acetate (4: 1), were combined and labelled as PEA-22, PEA-29, and PEA-48. 

*TLC and HPTLC analysis for β -sitoserol*

Isolated and dried compound obtained by column chromatography as CM-20, CM-24, HEA-2, and PE-34 were subjected to TLC in two different mobile phases Chloroform: methanol (15: 1) and Petroleum ether: ethyl acetate (4: 1) and the spots were detected by reagent Anisaldehyde-sulphuric acid β-sitosterol was used as working standard for quantification of content in extracts. HPTLC of std. β -sitosterol with chloroform and petroleum ether fractions were performed. 

Isolated and dried antitubercular compounds obtained by different column chromatography are CM-20, CM-24, HEA-2 and PE-34 was subjected to HPTLC analysis. 

*Spectral data*

The isolated Compound PE-34 was single component identified from HPTLC analysis and others were mixture of components. The structure of PE-34 was elucidated using spectroscopy methods including IR, 1H NMR, GC-MS and also the structures were confirmed by comparison against literature spectroscopic data (27, 28) 

## Results and Discussion

*Antitubercular activity*

The results of NRA and BacT/Alert methods revealed that 150 μg/mL of petroleum ether and chloroform fraction of ethanol extract of *Vitex negundo *Linn possess antitubercular activity while methanol fraction did not show any antitubercular activity. Hence these two fractions were used for separation and isolation of phytoconstituents by column chromatography method. 

From the results of TLC, Rf values of chloroform and petroleum ether fraction matcheto betulinic acid, ursolic acid, and β-sitosterol reported in literature (6). Therefore, column chromatography of both fractions and ethanol extract were carried out by using different proportions of solvents. A total of 14 compounds isolated from different column chromatography and antitubercular activity were performed by NRA.

**Table 1:** Effects of isolated compounds on *M. tuberculosis *by NRA method

**Sr. no.**	**Name of the isolated compounds**	**Concentration/MIC (µg/mL)**	**Growth of ** ***M. tuberculosis *** **indicated by reddish/violet colouration**		
			**I**	**II**	**III**
1	Control without drug	--	+	+	+
2	Rifampicin	40	**--**	**--**	**--**
3	Isoniazid	0.2	**--**	**--**	**--**
4	Compound HEA-1	50	+	+	+
		100	+	+	+
5	Compound HEA-2	50	+	+	+
		100	**--**	**--**	**--**
6	Compound CM-20	50	+	+	+
		100	**--**	**--**	**--**
7	Compound CM-24	50	+	+	+
		100	**--**	**--**	**--**
8	Compound PE-9	50	+	+	+
		100	+	+	+
9	Compound PE-10	50	+	+	+
		100	+	+	+
10	Compound PE-19	50	+	+	+
		100	+	+	+
11	Compound PE-34	50	**--**	**--**	**--**
		100	**--**	**--**	**--**
12	Compound PEA-22	50	+	+	+
		100	+	+	+
13	Compound PEA-29	50	+	+	+
		100	+	+	+
14	Compound PEA-48	50	+	+	+
		100	+	+	+

The results revealed that HEA-2, CM-20, CM-24, and PE-34 inhibit growth of *M. tuberculosis* which interpreted as –ve NRA means possessing antitubercular activity at 100 µg/mL while PE-34 at 50 and 100 µg/mL.

**Figure 1:**
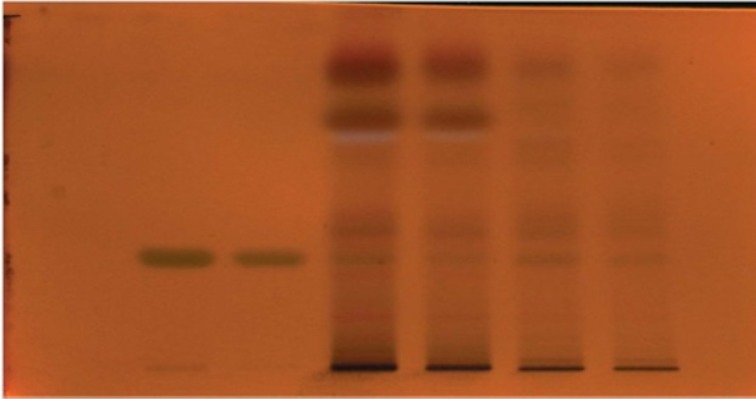
HPTLC image of std. β-sitosterol with chloroform fraction and petroleum ether fraction at 574 nm

**Table 2:** Identification and estimation of β -sitosterol by HPTLC

**Sr. no.**	**Sample name**	**Rf value**	**Area**	**β -sitosterol content (%w/w)**
1	Pet. ether fraction	0.28	2492.8	6.463
2	Chloroform fraction	0.32	2260.9	5.862
3	Std. β-sitosterol	0.29	7713.1	---

**Figure 2:**
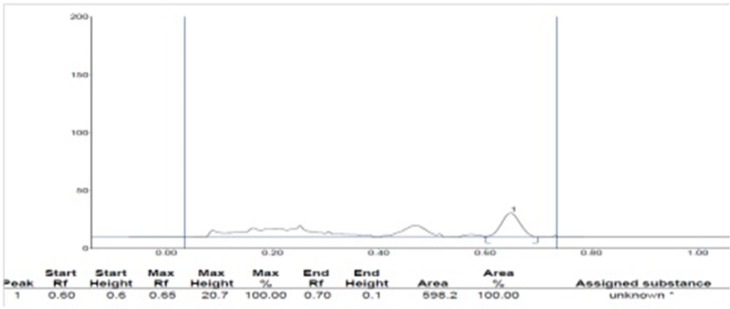
HPTLC study of isolated PE-34 (Ursolic acid)

*Spectral data*

Ursolic acid: Molecular formula C_30_H_48_O_3, _Molecular wt. 456.70032g/mol. white amorphous powder (50mg) Rf 0.65 (TLC and HPTLC , petroleum ether :ethyl acetate 4:1). IRνmax cm-1: 3450(free-OH), 2923(= C-H) 2852(C-H), 1709(C = O), 1657(C = C), 1384.64 (C-O) 1H NMR (300 MHz, CDCl3): δH 5.575 ppm(1H,s,H-12), δH 4.15(OH,s,H-3), δH 3.569 (2H,s,H-1), δH 3.569 (2H,s,H-3), δH 2.095 (2H,s,H-1), δH 2.095 (2H,s,H-18), δH 1.030 (CH_3_,s,H-27), δH 0.826(CH_3_,m,H-23), δH 0.851(CH_3_,m,H-24), δH 0.877(CH_3_,m,H-25), δH 0.898(CH_3_,m,H-26),δH 0.899(CH_3_,m,H-29), 0.899(CH_3_,m,H-30),δH1.217(CH_2_,m,H-2), δH1.251(CH_2_,m,H-3),δH1.284(CH_2_,m,H-5), δH 1.298(CH_2_,m,H-6), δH 1.298(CH_2_,m,H-7), δH1.420(CH_2_,m,H-21), δH 1.427(CH_2_,m,H-22), δH 1.459(CH_2_,s,H-28), δH 1.603(CH_2_,s,H-29) and δH 11.20(COOH,s,H-1).

**Figure 3:**
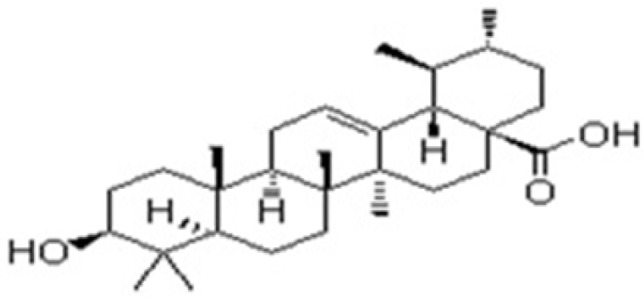
The chemical Structure of ursolic acid isolated from *Vitex negundo *Linn

**Figure 4:**
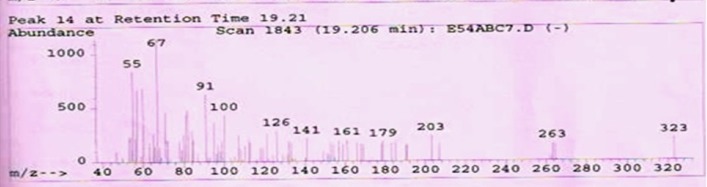
GC-MS analysis (*rel.int*%):*m/z*

**Table 3:** Fragmentation pattern of isolated ursolic acid by GC-MS from plant

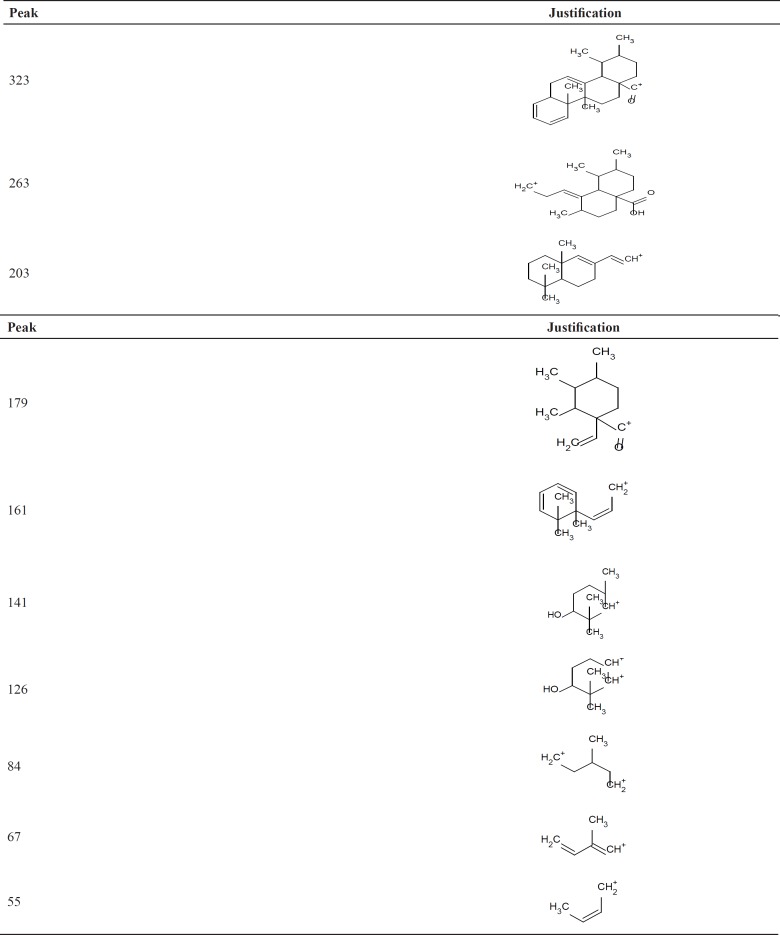

The results of isolated PE -34 were in good agreement with those obtained from IR, 1H-NMR, GC-MS spectral data.

## Conclusion

Ursolic acid showed a remarkable antitubercular activity against *Mycobacteria tuberculosis* H37RV. More definite studies *in-viv*o were required to define the mechanism of action of these compounds and elucidate compounds responsible for antitubercular activity on *Vitex negundo* Linn. in future.
